# Scale-Up of Academic Mesenchymal Stromal Cell Production

**DOI:** 10.3390/jcm12134414

**Published:** 2023-06-30

**Authors:** Caroline Laroye, Mélanie Gauthier, Jessica Morello, Naceur Charif, Véronique Latger Cannard, Céline Bonnet, Alain Lozniewski, Andrei Tchirkov, Natalia De Isla, Véronique Decot, Loïc Reppel, Danièle Bensoussan

**Affiliations:** 1 CHRU Nancy, Cell Therapy and Tissue Bank Unit, MTInov Bioproduction and Biotherapy Integrator, F-54000 Nancy, France; m.gauthier@chru-nanacy.fr (M.G.); j.morello@chru-nancy.fr (J.M.); v.decot@chru-nancy.fr (V.D.); l.reppel@chru-nancy.fr (L.R.); d.bensoussan@chru-nancy.fr (D.B.); 2 CNRS, IMoPA, Lorraine University, F-54000 Nancy, France; naceur.charif@univ-lorraine.fr (N.C.); natalia.de-isla@univ-lorraine.fr (N.D.I.); 3 CHRU Nancy, Flow Cytometry Platform, Hematology Laboratory, F-54000 Nancy, France; v.cannard@chru-nancy.fr; 4 CHRU Nancy, Genetics Laboratory, F-54000 Nancy, France; c.bonnet@chru-nancy.fr; 5 CHRU Nancy, Department of Microbiology, F-54000 Nancy, France; a.lozniewski@chru-nancy.fr; 6 CHRU Clermont-Ferrand, Medical Cytogenetics Laboratory, F-63003 Clermont-Ferrand, France; a.tchirkov@chu-clermontferrand.fr

**Keywords:** mesenchymal stem cells, Wharton’s jelly, scale-up

## Abstract

Background: Many clinical trials have reported the use of mesenchymal stromal cells (MSCs) following the indication of severe SARS-CoV-2 infection. However, in the COVID19 pandemic context, academic laboratories had to adapt a production process to obtain MSCs in a very short time. Production processes, especially freezing/thawing cycles, or culture medium have impacts on MSC properties. We evaluated the impact of an intermediate cryopreservation state during MSC culture to increase production yields. Methods: Seven Wharton’s jelly (WJ)-MSC batches generated from seven different umbilical cords with only one cryopreservation step and 13 WJ-MSC batches produced with intermediate freezing were formed according to good manufacturing practices. The identity (phenotype and clonogenic capacities), safety (karyotype, telomerase activity, sterility, and donor qualification), and functionality (viability, mixed lymphocyte reaction) were analyzed. Results: No significant differences between MSC production processes were observed, except for the clonogenic capacity, which was decreased, although it always remained above our specifications. Conclusions: Intermediate cryopreservation allows an increase in the production yield and has little impact on the basic characteristics of MSCs.

## 1. Introduction

Since December 2019 and the occurrence of SARS-CoV-2 in China, pharmaceutical and academic laboratories have performed intense research to find effective treatments for COVID-19. Among potential therapeutics, mesenchymal stromal cells (MSCs) appear to be promising candidates, as they have previously provided beneficial effects for similar indications, such as sepsis, acute respiratory distress syndrome (ARDS), and septic shock, while exhibiting a favorable safety profile, as reported in a meta-analysis including 55 studies [1]. Indeed, MSC injection in preclinical models of sepsis and septic shock improves organ failure, decreases plasma and tissue inflammation, increases bacterial clearance, and finally, improves survival [2,3,4]. Similarly, a protective effect of MSCs on viral sepsis [5,6]. MSCs exhibit different properties that are helpful for ARDS, including (i) a proangiogenic effect [7] and an antiapoptotic effect on endothelial cells [8], (ii) the restoration of alveolar fluid clearance thanks to angiopoietin 1 and hepatocyte growth factor secretion and the protection of tight junctions [9], and also (iii) an anti-inflammatory effect through the shift of alveolar macrophages to the M2 phenotype thanks to MSC mitochondrial transfer [10], macrophage efferocytosis of apoptotic MSCs [11], and/or the paracrine effects of MSCs [12]. A report of the first compassionate use of MSCs in a Chinese patient with severe SARS-CoV-2 infection was published in January 2019 [13,14]. The promising results of this study have led to many clinical trials evaluating one or repeated infusions of MSCs themselves under the status of advanced therapy drugs [14,15,16] or using MSCs as raw material for extracellular vesicle (EV) production [17,18].

However, most of those clinical trials were sponsored by academic institutions with MSC production mainly supported by academic laboratories. However, the COVID-19 crisis highlighted their limits. Indeed, the need to produce a large number of MSCs in a short time required adaptations from cell therapy units. First, the accessibility of MSC sources became a major criterion, which led cell therapy units to favor a fetal source, such as Wharton’s jelly, over adult sources, such as bone marrow or adipose tissue [19]. The abundance, ease of access, and lack of health restrictions regarding collection and the expansion capacity of fetal MSCs, which are more immature, are indisputable advantages to obtaining enough raw material.

However, to reach the number of cells usually infused in clinical trials (70 to 90 × 10^6^ MSCs/patient/dose, patients receiving one dose or repeated infusions [20]), MSC production requires an expansion time of several weeks, which was not compatible with the health emergency implied by COVID-19. To shorten this time, secondary to the French regulatory agency’s authorization, we decided to freeze the production surplus obtained at early passage (P2) and thaw them later to generate other cell batches. Therefore, the incompressible time of around one month required by the first passage (P0) only affected the first batch. However, this strategy needs two cycles of cell freezing: the first in the early passages and the second at the end of production to obtain off-the-shelf cells.

Nevertheless, the impact of repeated freeze/thaw cycles on the characteristics of MSCs is currently still controversial. Some studies have highlighted deleterious effects of cryopreservation on MSC immunomodulatory properties, while others did not observe any impact [21,22]. If freezing the final therapeutic product is the only way to use MSCs in vital emergency indications and for the industrialization of MSC production, the balance between advantages and drawbacks provided by intermediate cryopreservation remains unclear. Intermediate cryopreservation reduces the cost and time of production but could impair the MSC production quality. However, Cottle et al. reported that performing one or more freezing cycles during the production of MSCs from bone marrow has the same impact on cell quality [22].

One of the pandemic consequences was the shortage in reagent and consumable production or even their discontinuation from marketing, compelling production units to have process validations with several reagents, consumables, or types of equipment.

In this study, we compared two methods of large-scale GMP-compliant expansion of MSCs from Wharton’s jelly (WJ-MSCs) in the context of different clinical trials. In the first, “continuous production”, only one cryopreservation procedure was performed. In the second, “discontinuous production”, intermediate freezing was performed at the end of passage 2. This second strategy allowed our academic cell therapy unit to produce enough cells in a short time to treat 15 patients experiencing severe COVID-19 infection in a phase II clinical trial (EudraCT No: 2020-002772-12). Finally, we compared the production of MSCs cultivated with two different platelet lysates.

## 2. Materials and Methods

### 2.1. GMP-Compliant Isolation and Expansion of MSCs

Umbilical cords were collected at the Maternity Unit of Nancy University Hospital. Before collection, pregnant mothers signed an informed consent in compliance with the French national regulations regarding human sample collection, manipulation, and personal data protection. This collection was approved by the Nancy University Hospital ethics committee and the French Ministry of Research (No. AC-2021-4733). For 1 h, the umbilical cords were immersed in an antibiotic–antifungal solution (amphotericin B (0.05 g/L); vancomycin (1 g/L); amoxicillin (1 g/L)) and then cut into 5 cm pieces. Each section was placed in a small flask (TPP, Trasadingen Switzerland) for 15 min for adhesion to the plastic surface. Afterwards, 30 mL of αMEM medium (Macopharma, Mouvaux, France) supplemented with 5% of MultiPL30i platelet lysate (Macopharma, Mouvaux, France) was added. The culture was carried out at 37 °C and under hypoxic conditions (5% of O_2_ and 5% of CO_2_). After 4 to 5 days of culture, the medium was changed. Once cells had migrated from the umbilical cord and adhered to the plastic (about 10 days), sections of cords were removed, and the medium was renewed. When 80% cell confluence was obtained, the medium was removed, small flasks were washed with PBS (Macopharma, Mouvaux, France), and trypsinization was performed (TrypLE™ Select CTS™ (1×) Gibco, Grand Island, NE, USA). Cells were washed by centrifugation and plated using closed system kits (Macopharma, Mouvaux, France) into cellstacks for passage 1 (P1) (area surface 1272 cm^2^) (Corning, New York, NY, USA) at a density of 1000 MSCs/cm^2^.

### 2.2. Continuous Production (CP)

CP was performed as previously described [23]. MSCs were cultured until passage 3 (P3) and frozen in a cryopreservation solution, previously cooled to 4 °C, composed of 80% albumin (Vialebex, LFB, Les Ulis, France) and 20% dimethyl sulfoxide (DMSO) (WAK-Chemie Medical GmbH, Steinbach, Germany)). The cryopreservation solution was added in an equal volume to the cell suspension. Freezing was performed through a controlled-rate freezer (Planer Kryo, Middlesex, UK). MSCs were stored in vapor-phase nitrogen until thawing. In parallel, we sought to validate the new platelet lysate MultiPL100i (Macopharma, Mouvaux, France) in order to anticipate the withdrawal from the market of MultiPL30i. We performed a comparison between the standard 5% MultiPL30i and MultiPL100i at different percentages (2%, 3%, 4% and 5%) as a medium supplement in terms of the cell viability, cell proliferation, immunophenotype, and clonogenicity from P1 to P3.

### 2.3. Discontinuous Production (DP)

**Figure 1:**
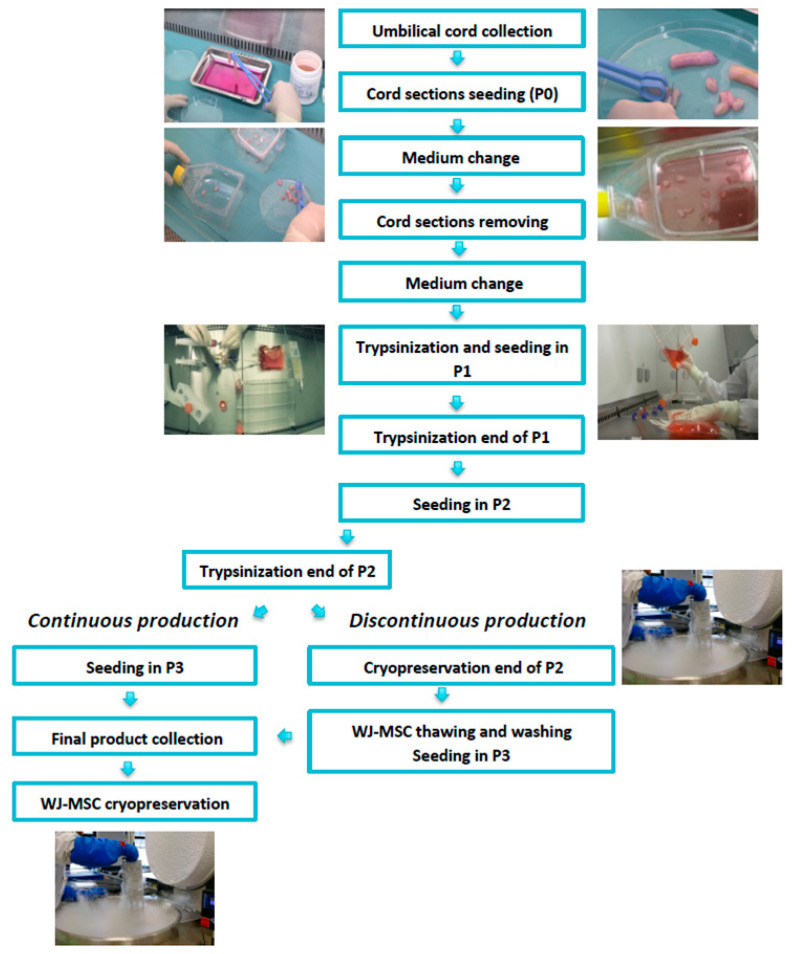
MSC manufacturing process. After MSC isolation from Wharton’s jelly by the explant method, cells were expanded until passage 2. At the end of passage 2, after trypsinization, the cells were either seeded in passage 3 (continuous production) or cryopreserved for further amplification (discontinuous production).

MSCs were cultured until passage 2 (P2), frozen in a cryopreservation solution, and stored in vapor-phase nitrogen. To obtain a new cell batch, MSCs in P2 were thawed in a water bath at 40 °C for 2–3 min and then washed with a solution composed of 50% albumin (Vialebex 4%) and 50% NaCl, on a SEPAX device (Cytiva, Marlborough, MA, USA). Using closed system kits (Macopharma, Mouvaux, France), MSCs were seeded into cellstacks (Corning, New York, NY, USA) at a density of 1000 MSCs/cm^2^ for P3. At the end of P3, MSCs were frozen using the same procedure as for CP (Figure 1).

The production was performed according to the French regulatory agency ANSM under approval number ETI/19/R/003.

### 2.4. Environmental Monitoring

Environmental monitoring was performed during MSC production. Nonviable 0.5 and 5 μm particle counts in an area of 1 m^3^ were monitored by an ApexZ50 particle counter (Lighthouse, White City, OR, USA). Viable particles were controlled by sedimentation plates every 4 h. The operators’ gloves were checked by five foot printings per glove at the end of production. Laboratory surfaces were monitored every week by the environmental biology laboratory of Nancy University Hospital.

### 2.5. MSC Quality Controls

#### 2.5.1. Infectious Markers

Biological parturient screening was performed in compliance with the regulatory infectious markers for any haematopoietic stem cell donation, including a viral genomic diagnosis for HIV, HBV, and HCV. Screening must be negative for HIV, HBV, HCV, HTLV, and syphilis. For EBV, CMV, and toxoplasmosis, only IgG antibodies can be positive.

#### 2.5.2. Microbiological Controls

Sterility assays were performed according to the European Pharmacopeia 10th edition. Aerobic and anaerobic bacterial blood culture BacT/ALERT^®^ tests (Biomérieux, Craponne, France) were used and analyzed on the BacT/ALERT® lecturer (Biomérieux, Craponne, France). They were seeded after collection, each time the medium was changed, at the trypsinization steps, and before freezing. These controls were performed by the Microbiology Laboratory of Nancy University Hospital.

### 2.6. MSC Characterization According to the ISCT Guidelines [24]

#### Cell Count and Immunophenotype Analysis

After each trypsinization step, an MSC count was performed by flow cytometry (MACSQuant10, Miltenyi Biotec, Bergisch Gladbach, Germany). The proliferation potential of WJ-MSCs during monolayer expansion was determined by population doubling (PD) according to the formula PD = [log10(NH) − log10(NI)]/log10(2), where NH is the harvested cell number and NI is the inoculum cell number, and by the doubling time (DT), according to the formula DT = T/PD, where T is the time between two passages in hours. The cumulative PD was calculated by adding the PD for each passage to the PD of the previous passages.

To evaluate the expression of surface markers, 2 × 10^6^ MSCs were labeled with a positive cocktail of antibody-labeled mesenchymal markers after 15 min incubation and containing anti-CD90, CD73, and CD105 mAbs, and with a negative cocktail of antibodies containing anti-CD34, CD45, CD11b, CD19, and HLA-DR mAbs (Stemflow hMSC Analysis Kit, BD Biosciences, Franklin Lakes, NJ, USA), according to the International Society for Cellular Therapy (ISCT) minimal criteria and the WHO recommendations (WHO/BS/2019.2376) [24]. Viable cells were identified using the 7AAD marker of dead cells. The absence of contamination by endothelial cells was controlled by the endothelial marker CD144 for WJ-MSCs. Samples were acquired with BD FACSLyric using BD Diva software v9.0 (BD Biosciences, Franklin Lakes, NJ, USA).

### 2.7. Clonogenicity Assays

The clonogenic capacity was evaluated by the colony-forming-unit fibroblast (CFU-F) assay. MSCs were seeded in T25 flasks at 10 and 20 cells/cm^2^. They were cultured in complete medium for 10 days. Then, they were washed with PBS, fixed with ethanol, stained with Giemsa solution (Sigma-Aldrich, Saint-Louis, MO, USA), and rinsed with water. CFU-Fs of more than 50 cells were scored, and the percentage of clonogenicity was calculated according to the following formula: (CFU-F number/Seeded MSC number) × 100.

### 2.8. Mixed Lymphocyte Reaction (MLR)

The immunogenicity and immunomodulation capacities of MSCs were analyzed. For this purpose, lymphocyte proliferation tests were performed with a DELFIA^®^ Cell Proliferation Kit (Perkin Elmer, Waltham, MA, USA), as previously described [23]. Peripheral blood mononuclear cells (PBMCs) from healthy donors were isolated using a density gradient medium (MSL, Eurobio Scientific, Les Ulis, France) and centrifugation. A total of 1 × 10^5^ PBMCs from an allogeneic donor 1 (D1) were cultured for 3 days alone or in coculture with either

1 × 10^5^ allogeneic irradiated (25 Gy) PBMCs from a second donor (D2*), used as stimulating cells for positive control;

1 × 10^5^ MSCs* irradiated (25 Gy) to test MSC immunogenicity;

Or 1 × 10^5^ PBMCs from D2* irradiated (25 Gy) and 1 × 10^5^ MSCs to test the MSC immunomodulation capacity.

### 2.9. Mesodermic Differentiation

Osteogenic and adipogenic differentiation was performed according to the manufacturer’s instructions (Differentiation Media BulletKits, Lonza, Basel, Switzerland). To induce osteogenesis, WJ-MSCs were seeded at 3.1 × 10^3^ cells/cm^2^ in complete medium in a 12-well plate. After 24 h, Osteogenesis Induction Medium (Lonza) was added to the adherent cells; the medium was replaced twice per week, and differentiation was continued for 21 days. The MSC control consisted of cultivating the cells with only basal medium. At day 21, calcium mineralization was assessed by coloration with Alizarin Red (Sigma-Aldrich, Saint-Louis, MO, USA). For adipogenic differentiation, WJ-MSCs were seeded at 2.1 × 10^4^ cells/cm^2^ in complete medium in a 12-well plate. At 100% confluence, three cycles of induction/maintenance were performed. Each cycle consisted of feeding MSCs with supplemented Adipogenesis Induction Medium (Lonza) and culturing for 3 days, followed by 1 to 3 days of culture in supplemented Adipogenic Maintenance Medium (Lonza). After three complete cycles, WJ-MSCs were incubated with Adipogenic Maintenance Medium for 21 days; the medium was replaced twice a week. The MSC control consisted of cultivating the cells with Adipogenic Maintenance Medium only. After 21 days, staining with Oil Red O solution (Sigma-Aldrich, Saint-Louis, MO, USA) was performed to detect lipid droplets.

### 2.10. Karyotype and Telomerase Activity

WJ-MSC karyotyping was performed by the Genetics Laboratory of Nancy University Hospital. Mitosis was blocked in metaphase by adding colchicine to the cultured cells. Then, cells were subjected to hypotonic treatment and fixed with fresh Carnoy Fixative (3:1 ratio of ethanol:glacial acetic acid). Standard cytogenetic analysis was performed after GTG-banding. Twenty metaphases were analyzed. Telomerase activity was determined by qRT-PCR by the TRAP method (telomere repeat amplification protocol) at the Cytogenetics Laboratory of Clermont-Ferrand Hospital, France. The results of real-time RT-PCR are expressed as the normalized hTERT expression, i.e., the ratio between hTERT and ABL transcript numbers multiplied by 100. Samples with an absence of hTERT amplification and a transcript copy number of ABL > 10,000 were considered negative for hTERT expression (Table 1).

### 2.11. Senescence Assay

The senescence assay was only performed in the context of LP comparison. The Senescence β-Galactosidase Staining kit (Cell Signaling, Danvers, MA, USA) and Click-iT Plus EdU Alexa Fluor 594 Imaging Kit (Thermo Fisher Scientific, Waltham, MA, USA) were used together to assess senescence cell rates according to the manufacturer’s protocol after 4 days of culture. At least 15 microscopy images were used to determine the percentage of senescence-associated beta-galactosidase (SA-βgal) activity and EdU positive cells (Leica DMI3000B). Cells were considered senescent if they were positive for SA-βgal and negative for Edu.

### 2.12. Statistics

The unpaired and nonparametric Mann–Whitney test and ANOVA (one-way or two-way) with Bonferroni correction were used for the comparison of two groups or three or more groups, respectively. The Wilcoxon matched-pairs signed rank test was performed to compare continuous P3 and discontinuous P3 production. Measurements were summarized as the mean ± SD, and experimental sample numbers (n) are indicated in the Figure legends. Statistical significance was defined at *p* < 0.05. The statistical analysis was performed with Prism V.9 (GraphPad Software).

## 3. Results

### 3.1. Production Data from the MSC Manufacturing Process

**Figure 2:**
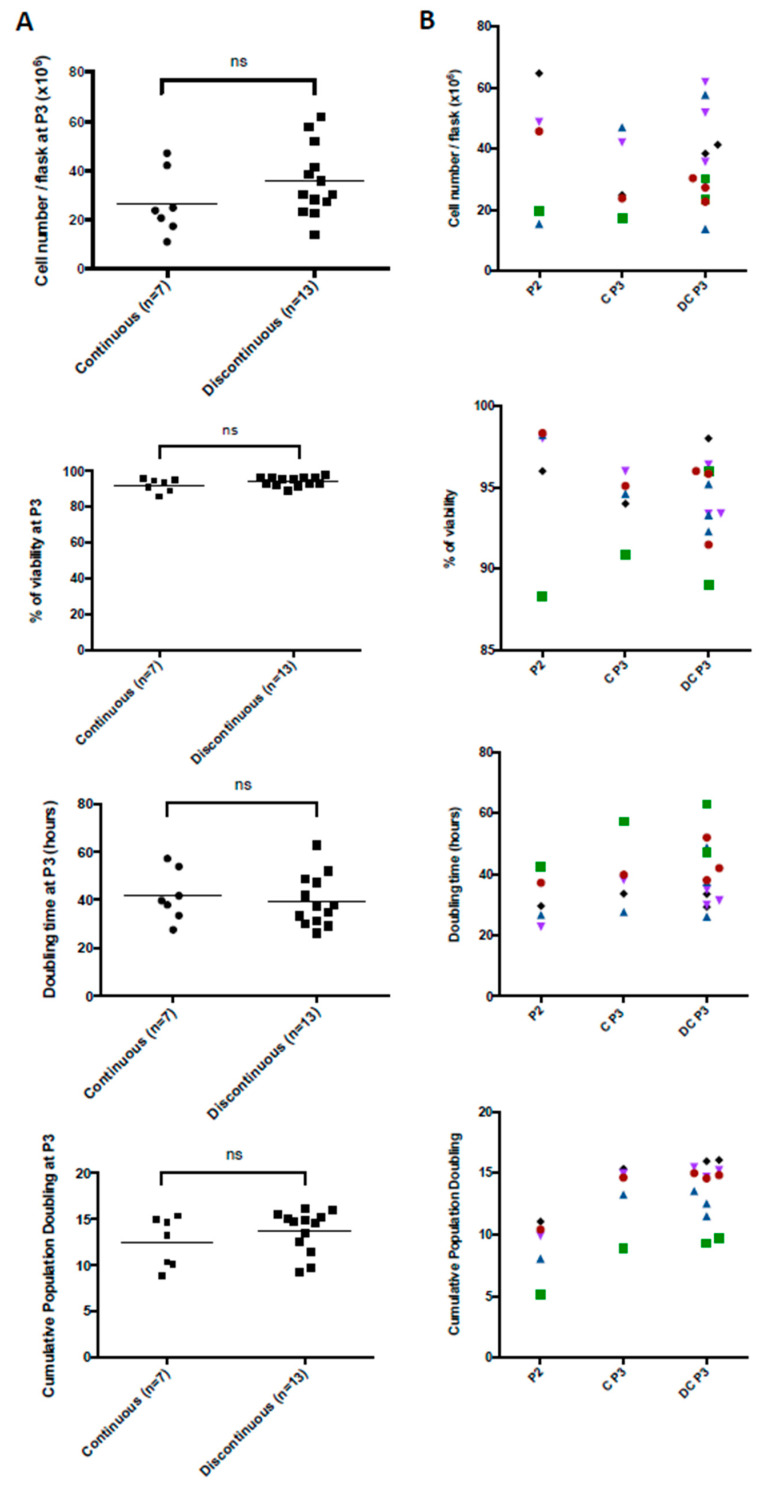
Production data from the MSC manufacturing process. (**A**) Seven continuous and thirteen discontinuous MSC production processes were analyzed at the end of P3 in terms of the cell number, viability, doubling time, and cumulative population doubling. (**B**) A paired analysis was also performed between continuous (n = 5) and discontinuous (n = 13) MSC production on the same parameters. (P2: passage 2, CP P3: continuous passage 3, DP P3: discontinuous passage 3, ns: not significant). One color symbol corresponds to one umbilical cord.

Overall, we analyzed seven continuous and thirteen discontinuous MSC production procedures. At the end of P3, regardless of the production mode, no significant difference was noticed in terms of the cell number per flask (26.7 ± 13.1 × 10^6^ vs. 35.6 ± 14.3 × 10^6^), viability (92.2 ± 3.7% vs. 94.3 ± 2.5%), DT (41.7 ± 10.6 h vs. 39.5 ± 10.6 h), or cumulative PD (12.5 ± 2.7 vs. 13.7 ± 2.3) (Figure 2A). Through a paired analysis, we showed no significant difference between continuous and discontinuous P3 production starting from the same P2 (Figure 2B). The intermediate freezing/thawing step seems to have no negative impact on production data, and MSCs exhibited a very good recovery upon reculture. Moreover, at the end of P3, from each UC, we obtained around eight to twelve treatment doses with DP compared to two to three with CP.

### 3.2. Particle Controls in the Production Environment

The particle content was determined during the different stages of MSC production. No significant differences were observed regarding the number of nonviable 0.5 and 5 μm particles measured under hoods at any stage of production (Figure S1A). However, significant differences were observed between the final stages of production (end of P3) and the early stages (P0 and end of P0) regarding the number of nonviable 0.5 and 5 μm particles found in the production room (Figure S1B). These differences are the consequence of longer final steps, requiring the presence of an additional person in the room and for more material to be used. It should be noted that no significant differences were observed between the stages representing DP (thawing and seeding in P3) and CP (end of P2 and seeding in P3).

### 3.3. Quality Control Data from the MSC Manufacturing Process

**Figure 3:**
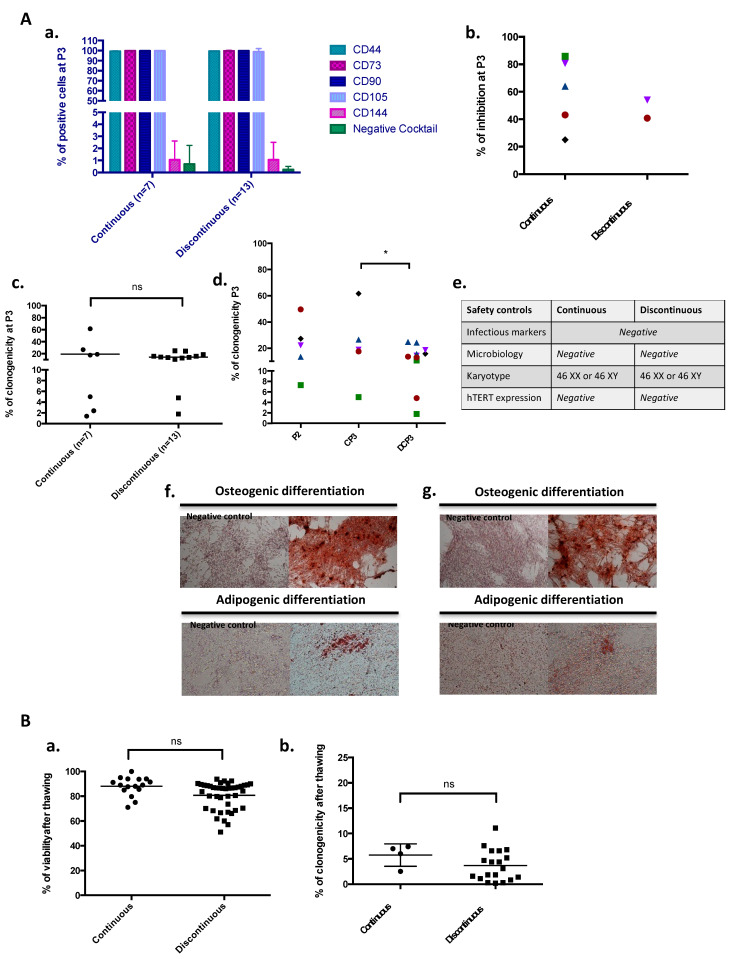
Quality control data from the MSC manufacturing process. (**Aa**) Immunophenotype analysis at the end of P3 after continuous (n = 7) and discontinuous (n = 13) MSC production. Data represent the mean ± SD. (**Ab**) Immunomodulation capacity of MSCs at P3 between continuous (n = 5) and discontinuous (n = 2) MSC production. (**Ac**) The clonogenic capacity was evaluated by the colony-forming-unit fibroblast (CFU-F) assay at the end of P3 after continuous (n = 7) and discontinuous (n = 13) MSC production. (**Ad**) A paired analysis was also performed between continuous P3 (n = 5) and discontinuous P3 (n = 13) production starting from the same P2 (* *p* < 0.05, CP P3 vs. DP P3). (**Ae**) Safety controls were performed at the end of P3 after continuous (n = 7) and discontinuous (n = 13) MSC production. (**Af**,**Ag**) Osteogenic and adipogenic differentiation capacities were evaluated at the end of P3 after continuous (**Af**) and discontinuous (**Ag**) MSC production by the revelation of calcium deposits and lipid vesicles, respectively. (**Ba**) Viability after thawing continuous (n = 16) and discontinuous (n = 42) MSC production. (**Bb**) The clonogenic capacity evaluated by the CFU-F assay after thawing continuous (n = 4) and discontinuous (n = 19) MSC production. (P2: passage 2, CP P3: continuous passage 3, DP P3: discontinuous passage 3, ns: not significant). One color symbol corresponds to one umbilical cord.

Regarding quality control data from the MSC manufacturing process, the MSC identity, functionality, and safety were evaluated throughout production. MSCs steadily expressed CD44, CD73, CD90, and CD105 mesenchymal markers (above 98%) and were considered negative for haematopoietic markers and the endothelial marker CD144 (below 5%) at every step of production, regardless of the production mode (Figure 3(Aa)). At the end of P3, after CP or DP, no significant difference was shown in terms of the MSC clonogenic capacity (19.1 ± 21% vs. 14.2 ± 6.7%, always above our specification) (unpaired test) (Figure 3(Ac)). However, the Wilcoxon matched-pairs test highlighted a significant decrease in MSC clonogenicity after DP compared to CP (*p* = 0.0122) (Figure 3(Ad)). MSCs exhibited an immunomodulation capacity, inhibiting allogeneic reaction at 59.7 ± 25.6% (n = 5) and 47.4 ± 9.3% (n = 2) after CP and DP, respectively (Figure 3(Ab)). The low MSC immunogenicity was observed for both production modes through the proliferation of PBMCs from a healthy donor in coculture with MSCs below 10%. The mesodermic differentiation capacity was confirmed at the end of CP (Figure 3(Af)) and DP (Figure 3(Ag)) through osteogenic and adipogenic differentiation. After 21 days in contact with a specific differentiation medium, after both CP and DP, MSCs showed osteogenic and adipogenic differentiation capacities by the revelation of calcium deposits and lipid vesicles, respectively. Infectious markers from the donors were all negative. No microbiological contamination of the product was found during the MSC manufacturing process. Whatever the production mode, the analysis of the final-product karyotypes before freezing at the end of P3 showed the absence of aneuploidy, and telomerase activity was never detected (Figure 3(Ae)). As a conclusion, the good cell recovery after the intermediate freezing/thawing step in the DP mode was also confirmed by most of the quality controls. Moreover, we performed some controls after the thawing of CP and DP MSC production and did not observe any significant differences regarding the phenotype, viability (Figure 3(Ba)) or clonogenic capacity (Figure 3(Bb)).

### 3.4. Comparison between MultiPL30i and MultiPL100i

**Figure 4:**
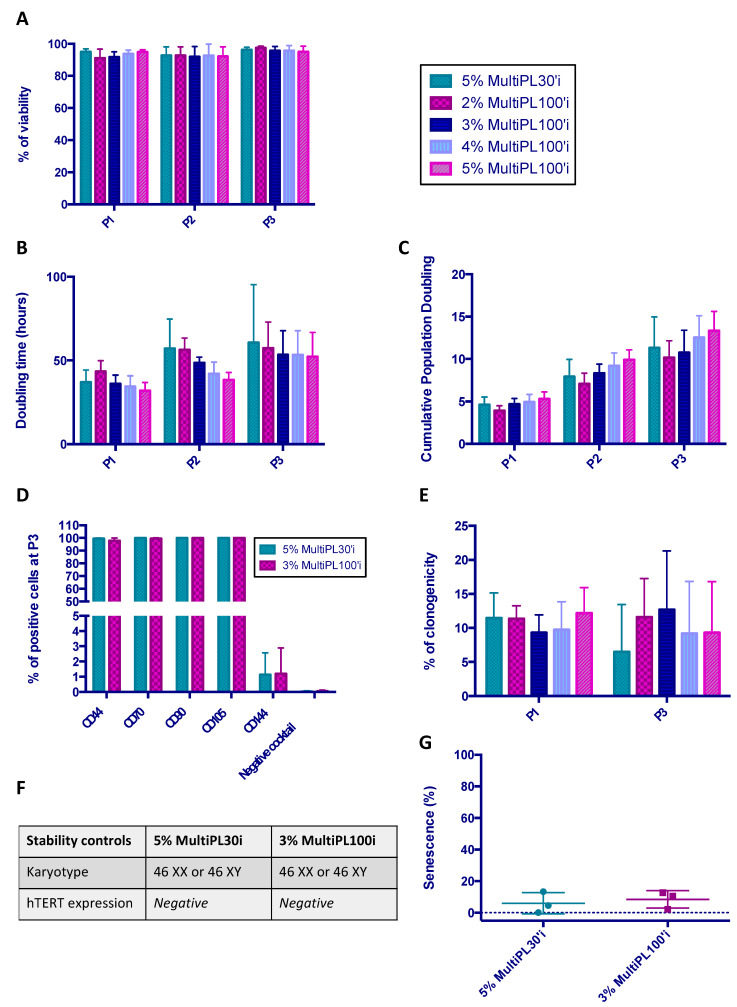
Comparison between MultiPL30i and MultiPL100i. A comparison between the standard 5% MultiPL30i and MultiPL100i at different percentages (2, 3, 4, and 5%) as a media supplement in terms of cell viability (**A**), cell proliferation (**B**,**C**), immunophenotype (**D**), and clonogenicity (**E**) from P1 to P3 was performed. At the end of P3, additional testing for stability (karyotype and telomerase activity) (**F**) and senescence assays (**G**) were implemented to compare MultiPL30i and MultiPL100i. (**G**) Percentage of senescent cells. Data represent the mean ± SD (n = 3).

A comparison between the standard 5% MultiPL30i and MultiPL100i at different percentages (2%, 3%, 4%, and 5%) as a medium supplement in terms of the cell viability, cell proliferation, immunophenotype, and clonogenicity from P1 to P3 was performed. At the end of P3, additional testing for stability (karyotype and telomerase activity) and senescence assays was implemented to compare MultiPL30i and MultiPL100i. We showed that, regardless of the percentage of MultiPL100i used, the MSC viability was always above 90% during production (Figure 4A). According to the proliferation data (DT and cumulative PD), even when no significant difference was observed, 3% MultiPL100i seemed to exhibit similar proliferation data to the standard 5% MultiPL30i. Higher MultiPL100i concentrations showed faster cell proliferation, mostly at P1 and P2 (Figure 4B,C). MultiPL100i supplementation in MSC culture media showed no impact on the MSC immunophenotype. MSCs steadily expressed mesenchymal markers (around 100%) and were considered to be negative for haematopoietic markers and the endothelial marker CD144 (below 5%) (Figure 4D). Unlike what is usually observed with LP30i, we reported, only at the end of P0, the presence of adherent cells similar to monocytes when WJ-MSCs were expanded in the presence of LP100i. The flow cytometry analysis performed after the detachment of WJ-MSCs did not show any differences between cells cultivated in P0 with LP30i and those cultivated with LP100i, especially in the absence of haematopoietic markers using the negative cocktail. The MSC clonogenic capacity was maintained with MultiPL100i, regardless of the concentrations at P1 and P3 (Figure 4E). Whatever MultiPL was used, the analysis of the final product at the end of P3 showed the absence of aneuploidy (Figure 4F), telomerase activity was never detected (Figure 4F), and the percentage of senescent cells remained very low (means < 10%) (Figure 4G).

## 4. Discussion

In this study, we report the implementation, during the COVID-19 pandemic, of a strategy to improve the yield of WJ-MSC production in a short time, allowing fast patient inclusion in an academic clinical trial, considering the high frequency of patients during each wave. To shorten the production time, it was decided to freeze the production surplus obtained at the early passage (P2) and thaw them later to generate other cell batches (P3). Through this study, we demonstrated that intermediate freezing did not affect the basic and functional characteristics of WJ-MSCs, except for the clonogenic capacity, which was decreased, although always remaining above our specifications. Considering the microbiological contamination risk, we report no difference between the two production modes. Finally, the Master and Working Cell Bank strategy allowed an academic laboratory to produce the number of cell doses required in a short time (6 months), allowing the inclusion of patients in a clinical trial sponsored by Nancy University Hospital on ARDS secondary to SARS-CoV-2 infection (EudraCT No: 2020-002772-12), while reducing the production costs. Indeed, the cost of WJ-MSCs at the end of P3 was calculated for CP and DP processes considering reagents and consumables, technical and pharmaceutical staff, quality controls, and environmental controls. A 25% decrease in the cost was observed with DP and was consistent with a previous study that highlighted the interest in the Master and Working Cell Bank strategy in terms of its space-, time- and cost-efficient manner to produce MSCs for clinical trials [25].

However, in many academic trials initiated with MSCs for this indication at the same time, the number of patients enrolled was low or the MSC dose was suboptimal, a factor that was certainly limited by the MSC production capacity of the cell therapy units (among nearly sixty clinical trials developed on COVID-19 ARDS all over the world in June 2020, fewer than five treated more than forty patients). Such an observation is probably part of the explanation for the absence of significant results in different comparative phase II clinical trials [26]. If the MSC dose is suboptimal or if the number of enrolled patients is underestimated, a promising therapy could be improperly ruled out. Therefore, there is a real challenge to improve the yield of MSC production while decreasing the cost. This is why our study paves the way for the scale-up of the MSC production process. However, although we report an improvement of the rate of MSC production in cellstacks with the DP mode, this may not be enough to carry out large-scale phase II or III clinical trials.

Indeed, if biomedicines are the drugs of the near future, it is necessary to change the production process to dramatically improve the yield. One way to reach this goal is to perform MSC expansion in a stirred and online-monitored bioreactor using microcarriers for MSC adhesion while regularly perfusing fresh culture medium and empty microcarriers to allow expansion by bead-to-bead transfer [27].

Moreover, it is relevant to standardize MSC production. Currently, bioproduction is critically dependent on the suppliers of kits, reagents, and culture media. As mentioned above, all the MSC production reported in this study was performed using LP30i. However, its discontinuation from the market compelled us to validate the use of LP100i. Platelet lysate is one example among many others. Bags, kits, reagents, and media are withdrawn from the market according to regulatory developments and changes in company business strategies, leading production units to constantly adapt or even perform clinical trials with cells produced differently at the beginning and the end. Can we really consider cells to be similar if they have not been produced under the same conditions? In addition to MSC sources or the characteristics of the donors, which are widely described as affecting MSC properties, cell culture conditions affect the final product [23,28,29]. Hypoxia, confluence, seeding density, and passage influence the MSC capacity [30,31,32]. Regarding the medium’s impact, we report that the basic MSC properties were not modified whether they were produced with LP100i or LP30i. The composition of platelet lysates has been reported to be impacted by their production process. For example, Shanbhag et al. recently reported that the duration of platelet concentrate freezing significantly affects the concentrations of cytokines, chemokines, and growth factors from produced platelet lysates [33]. Many studies have reported that the different MSC properties (immunomodulation, differentiation, proliferation, etc.) can be enhanced when MSCs are primed. The addition of IFNγ, IL1β, LPS, poly-IC, or TNFα considerably changes the profile of MSCs by notably directing them towards more or less immunomodulatory or immunosuppressive phenotypes [34,35]. However, the composition of platelet lysates or ready-to-use media is unknown and remains the property of the manufacturers. Therefore, it is hardly achievable for cell production units to ensure the reproducibility of MSC production from batch to batch, unless dedicating the same batch of each reagent to a whole clinical trial. However, this limits comparisons between clinical trials, unless a standardized potency assay is developed for each MSC indication or property. Compared to chemical drugs, it would seem quite incongruous for the manufacturer to not know the exact composition of an essential excipient for the production.

The production process reported in this study relies on two cryopreservation steps. We did not show any difference regarding the viability of WJ-MSCs at the end of P3, even with a matched-pairs analysis, although a cryopreservation step was introduced at P2 in the DP process. The only impairment observed in this study was in terms of the clonogenic capacity, which was significantly lower than with the continuous mode, although it was above our specification. If viability is not implicated, one could suggest that the apoptosis of MSCs is increased. Unfortunately, we did not study apoptosis. However, we wonder whether if this cryopreservation step affected apoptosis if it would really impact the efficacy. Several studies have demonstrated that apoptotic MSCs could prove to be effective. Sung et al. demonstrated that the administration of apoptotic MSCs from adipose tissue is more effective than that of live MSCs in a sepsis murine model. The authors noted an improvement in parenchymal kidney and lung injuries and decreases in inflammation, apoptosis, and greater oxidative stress after the administration of apoptotic MSCs [36]. Pang et al. recently demonstrated that the deletion of the apoptotic effectors BAX and BAK decreases the immunomodulatory capacity of MSCs [37]. They demonstrated a lower efficacy of apoptosis-resistant MSCs compared to control MSCs in autoimmune encephalitis and asthma mouse models. As demonstrated recently by Galleu et al. in a GVHD model, the immunomodulatory effect of MSCs relies on the phagocytosis and efferocytosis of apoptotic MSCs, resulting in the polarization of recipient macrophages into an immunomodulatory phenotype producing indoleamine 2,3-dioxygenase (IDO) [11,37,38]. In these studies, the apoptosis of MSCs results from their targeting by recipient cytotoxic cells. Interestingly, this effect was also reported when dead cells were infused into a patient [11]. Such a mechanism of action raises the question of whether the results of clinical trials with similar infused doses of MSCs displaying different degrees of viability can be compared. Defining MSC doses in terms of the number of total cells and nonviable cells would then undoubtedly be relevant. Conversely, in some clinical trials, the lack of efficacy of MSCs has been assigned to their low viability. For example, in the START clinical trial, the authors noted no efficacy of MSCs in the indication of ARDS and attributed this lack of action to a wide range of cell viabilities, from 36% to 85% [39]. However, it is essential to specify that severe ARDS is associated with profound immune alterations, especially a decrease in the phagocytic capacity of macrophages [40]. Thus, the inability of macrophages to perform the phagocytosis of apoptotic MSCs could also be a hypothesis to explain the lack of efficacy of MSCs in this study.

The constant increase in knowledge of WJ-MSCs’ mechanism of action will help to define the relevant criteria for the calculation of the dose that must be infused. Moreover, the standardization of production procedures is urgently requested to allow comparisons between different studies and will go through the structuration of academic production center networks working for (i) the optimization of production processes and (ii) an increase in demand to supplier companies, leading them to maintain their products on the market. The entry of pharmaceutical manufacturers into the WJ-MSC production market will strongly contribute to these goals being reached.

## 5. Conclusions

The COVID-19 pandemic required strong responsiveness by academic ATMP departments to manage the rate of patient inclusion during the different waves. In this study, we described the implementation of a manufacturing process in our center to increase the level of MSC production in a short time, allowing us to provide treatments in such a constrained situation. Indeed, the basic capacities of MSCs were not significantly affected, regardless of the culture conditions (continuous versus discontinuous cultures), except for the clonogenic capacity, which decreased during the discontinuous production. Identification of the main mechanism of MSC actions will help us to analyze whether this decrease is correlated with efficacy of MSCs.

However, the academic facility limits were reached with this new manufacturing process, and another type of scale-up now needs to be developed to provide enough cells for phase IIb/III clinical trials. The use of bioreactors seems to be suitable, especially stirred and online-monitored bioreactors, to further reduce costs. By making this upgrade, academic GMP departments will also be able to support start-ups to perform the clinical development of innovative ATMPs.

## Figures and Tables

**Table 1:** MSC quality controls.

**Continuous**	**Reception**	**P0**	**P1**	**P2**	**P3**	
	Donor infectious markers	Bacteriology CFU-F Cell phenotype Count/Viability			Bacteriology Cell phenotype Karyotype CFU-F Count/Viability Telomerase activity MLR Osteocyte and adipocyte differentiation (first 3 batches)	
**Discontinuous**	**Reception**	**P0**	**P1**	**P2**	**Cryopreservation/Thawing**	**P3**
	Donor infectious markers	Bacteriology CFU-F Cell phenotype Count/Viability			Bacteriology Count/Viability	Bacteriology Cell phenotype Karyotype CFU-F Count/Viability Telomerase activity MLR Osteocyte and adipocyte differentiation (first 3 batches)

## Data Availability

Data requests should be sent to the corresponding author. Data access must be approved by the French data protection authority, la CNIL. For further information, please see https://www.cnil.fr.

## Supplementary Materials

The following supporting information can be downloaded at: https://www.mdpi.com/article/10.3390/jcm12134414/s1, Figure S1. Particle controls performed during the production process.
