# Supplementary material for: Scale-Up of Academic Mesenchymal Stromal Cell Production
Source: J Clin Med. 2023 Jun 30;12(13):4414. doi: 10.3390/jcm12134414 (PMC10342966; doi:10.3390/jcm12134414)
Supplement: Supplementary file 1 [file jcm-12-04414-s001.zip › jcm-2430124-supplementary.pdf]

Journal of *Clinical Medicine*

MDPI

Supplementary data

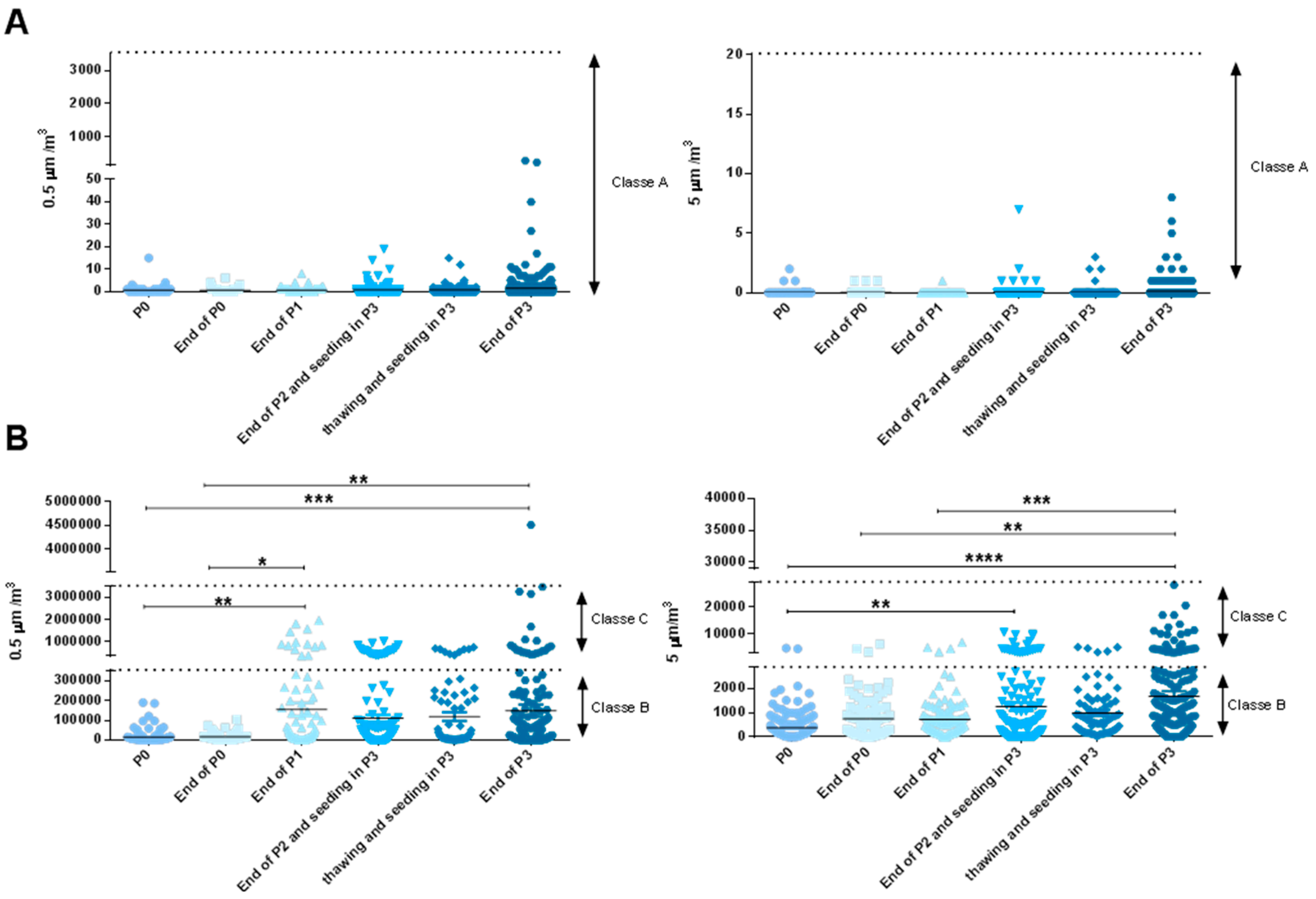

Figure S1. Particle controls performed during the production process. Particles of 0.5 µm and 5µm were measured continuously under the microbiological safety stations (A) and in the production room (B) during the different steps of the production process. The dotted lines correspond to the maximum allowed values according in French guidelines for ATMP production. Results are expressed as mean. *p<0.05; **p<0.01; ***p<0.001.
